# Imported Mollusks and Dissemination of Human Enteric Viruses

**DOI:** 10.3201/eid1606.091748

**Published:** 2010-06

**Authors:** David Polo, M. Luz Vilariño, Carmen F. Manso, Jesús L. Romalde

**Affiliations:** Universidad de Santiago de Compostela. Santiago de Compostela, Spain

**Keywords:** hepatitis A virus, norovirus, astrovirus, detection, imported mollusks, letter

**To the Editor:** The globalization of food production and trade has increased the potential risk for infectious foodborne diseases. Hepatitis A virus (HAV) and norovirus (NoV) constitute the most important foodborne pathogens of humans in terms of numbers of outbreaks and persons affected in industrialized countries (*1**,**2*). In these countries, improvement of health conditions and development of specific vaccines are changing the epidemiologic pattern of diseases such as hepatitis A, decreasing their prevalence and increasing the susceptibility of the unvaccinated adult population (*1*). In recent years, numerous cases of gastroenteritis caused by NoV and hepatitis A linked to imported shellfish have been reported (*2**–**5*). In Spain, 2 notable hepatitis A outbreaks associated with clams (*Donax* sp.) imported from Peru occurred in 1999 and 2008. In both situations, the Spanish Ministry of Health activated the National System of Epidemiologic Surveillance and the European Community Rapid Alert System for Foodstuffs. The implicated shellfish batches were immobilized or removed, and all the shellfish from Peru were banned from the European Union (*6*). We present further evidence that imported shellfish from developing countries, where these pathogens are endemic, can be a vehicle for viral gastroenteritis and HAV infections in areas where they are not endemic.

Fifty mollusk samples imported into Spain during September 2006–March 2009 were analyzed for NoV genotype I (GI) and GII, HAV and astrovirus (AsV). Countries of origin were Morocco, Peru, Vietnam, and South Korea (Table). The species studied were clams (*Callista chione,* n = 25; *Transanella pannosa*, n = 6; *Meretrix lyrata,* n = 3; and *Donax* sp., n = 5), oysters (*Crassostrea angulata,* n = 1), cockles (*Cerastoderma edule,* n = 1), and razor clams (*Solen marginatus,* n = 1 and *Ensis* sp., n = 8). Digestive tissue was dissected from duplicated samples (10–20 individual mollusks) and homogenized with 0.1% peptone water (pH 7.4), centrifuged at 1,000 × *g* for 5 min, and supernatant recovered. RNA was extracted by using both Total Quick RNA extraction Cells and Tissue kit (Talent, Trieste, Italy) and Nucleospin RNA Virus Kit (Macherey-Nagel, Düren, Germany).

**Table:** Viral detection and quantification in imported mollusk samples*

Country of origin (no. samples)	HAV positive†			Norovirus positive					Astrovirus positive, no. (%)¶
				GI‡			GII§		
	No. (%)	QR		No. (%)	QR		No. (%)	QR	
Morocco (34)				5 (15)	3.5 x 10^4^–1.1 x 10^7^		2 (6)	1.4 x 10^5^–8.9 x 10^5^	7 (21)
Peru (13)	1 (8)	4.7 x 10^3^		4 (31)	2 x 10^5^–1.8 x 10^7^		2 (15)	1 x 10^5^–1.4 × 10^6^	1 (8)
Vietnam (2)	1 (50)	4.4 x 10^7^		2 (100)	3.3 x 10^6^–7.7 x 10^7^				1 (50)
South Korea (1)				1 (100)	1.2 × 10^6^				–
Total (50)	2 (4)	10^3^–10^7^		12 (24)	10^4^–10^7^		4 (8)	10^5^–10^6^	9 (18)

*HAV, hepatitis A virus; GI, genotype I; QR, quantification range (RNA copies/g digestive tissue). †Obtained from *Donax* sp. and *Meretrix lyrata.* ‡Obtained from *Donax* sp., *Callista chione*, *Transanella pannosa*, *M. lyrata*, and *Ensis* sp. §Obtained from *Donax* sp., *T. pannosa*, and *Ensis* sp. ¶Obtained from *C. chione, T. pannosa, M. lyrata,* and *Ensis* sp.

NoV and HAV were detected by real-time reverse transcription–PCR (RT-PCR) by using the Platinum Quantitative RT-PCR Thermoscript 1-step system (Invitrogen, Carlsbad, CA, USA) (25 µL final volume) with 5 µL of template RNA, and primers, probes, and conditions as described (*7*). A sample that displayed a cycle threshold value <41 was considered positive. AsV was detected by standard RT-PCR (*7*), coupled with hybridization by using specific biotin-labeled probes with the commercial Kit Hybridowell universal (Argene, Varilhes, France).

Negative and specific positive controls for HAV, NoV, and AsV were introduced in each run. Real-time RT-PCR included appropriate external controls in each analysis to avoid underestimation of viral load. A mutant, nonvirulent, infective strain of mengovirus (vMC_0_) (10^3^ PFU) was used as control for extraction. To calculate the real-time RT-PCR efficiencies, external viral RNA (HAV, 10^3^ copies) or synthetic DNA (NoV, 10^5^ copies) controls for the respective virus were co-amplified with each template viral RNA as described (*8*). The number of RNA viral genome copies per gram of digestive tissue (RNA copies/g digestive tissue) was estimated by using standard curves generated from RNA transcripts and synthetic DNA (*8*) and corrected with the extraction and real-time RT-PCR efficiencies.

Twenty (40%) of 50 samples were contaminated by >1 virus (Table), although all the mollusk imports complied with the current sanitary standards. NoV GI was most prevalent, detected in 24% of samples, followed by AsV (18%), NoV GII (8%), and HAV (4%). One sample showed a low extraction efficiency (<1%), yielding all samples high real-time RT-PCR efficiencies (>10%) (*9*).

Six samples (30% of positive samples) were positive for >1 virus. Thus, 2 samples from Morocco showed contamination with NoV GI and AsV. From Peru, 1 sample was contaminated with both genogroups of NoV and another with NoV GI, NoV GII, and AsV. Samples from Vietnam (n = 2) were contaminated with HAV–NoV GI and NoV GI–AsV. Co-infection with different viruses or multiple virus strains could lead to more severe symptoms and the occurence of 2 episodes of the same or different diseases and also be a way to facilitate emergence of new recombinant strains (*10*).

Contamination levels for NoV GI ranged from 3.5 × 10^4^ to 7.7 × 10^7^ RNA copies/g digestive tissue; for NoV GII, from 1.03 × 10^5^ to 8.9 ×10^5^ RNA copies/g digestive tissue; and for HAV, from 4.7 × 10^3^ to 4.4 × 10^7^ RNA copies/g digestive tissue (Table). For HAV, these values are in the same range or even higher than in the coquina clams from Peru implicated in the outbreak in Spain in 2008, which is noteworthy because the attack rate for different batches of shellfish is dose dependent (*6*).

Determining the association of a viral infection with a particular contaminated product is often complicated, and the epidemiologic investigations necessary for finding this association are time consuming and allow the virus to spread before the problem is recognized. Furthermore, there are analytical difficulties in detecting and quantifying virus in shellfish samples and in monitoring them; other problems include ascertaining the representativeness of the sample (*2**,**6*) and the high cost of applying the technique in areas with extensive mollusk production.

The inadequacy of the bacterial indicators makes it necessary to develop new prevention strategies, based on microbial risk assessment, to ensure the sanitary quality of shellfish, both in production areas and in international trade operations. Implementing these methods and providing training to laboratories in developing countries are essential to achieving these objectives.
